# The Influence of Enterococcus faecalis as a Dental Root Canal Pathogen on Endodontic Treatment: A Systematic Review

**DOI:** 10.7759/cureus.7257

**Published:** 2020-03-13

**Authors:** Faisal Alghamdi, Marwa Shakir

**Affiliations:** 1 Oral Biology, King Abdulaziz University, Jeddah, SAU; 2 Endodontics, King Abdulaziz University, Jeddah, SAU

**Keywords:** endodontic treatment, infections, microbiology, enterococcus faecalis, bacteria, endodontic pathogens

## Abstract

Endodontic treatment failure may occur due to different causes such as persistence of bacteria, root canals that are poorly cleaned and obturated, improper coronal seal (leakage), and untreated canals (missed canals). The main reason for endodontic failure is the presence of some species of bacteria inside the root canal system such as Enterococcus (E.) faecalis. Those bacteria are more resistant to disinfection agents, causing a persistent intra-radicular or extra-radicular infection.

The current review aims to compile all the current studies concerning Enterococcus faecalis as a dental root canal pathogen that causes endodontic failure. In this systemic review, two databases, PubMed and Google Scholar, were searched using specific inclusion and exclusion criteria. Among 2943 studies, only 11 met the inclusion criteria and were included in the review for further analysis.

The 11 studies give prominence to the high distribution of Enterococcus faecalis within the root canal system. These studies investigated different aspects of Enterococcus faecalis, including its prevalence, resistance mechanisms, characteristics, express survival genes, and treatment.

The compiled data observed that most of the studies highlight Enterococcus faecalis as the primary pathogen associated with endodontic treatment. It has characteristic proprieties that make it capable of escaping disinfection means. Furthermore, clinical trials are required to examine E. faecalis and may provide valuable information about novel microbial detection methods to decrease the number of E. faecalis within the root canal system.

## Introduction and background

It is essential to remove all pulpal tissues, dentinal debris, and viable microorganisms from the root canal system during endodontic treatment. Since bacteria and their byproducts are the causative factors of pulpal and peri-radicular inflammation, their elimination is vital for successful endodontic treatment. Failure to effectively eliminate them could lead to persistent inflammation and impairment of healing [1]. Although proper canal instrumentation and adequate irrigation with sodium hypochlorite can decrease the number of bacteria, it cannot remove Enterococcus (E.) faecalis from the root canal entirely [1-2].

Enterococcus faecalis is an anaerobic gram-positive coccus that normally commences in the human oral cavity, gastrointestinal tract, and vagina because it has demonstrated good adaptation to such environments with rich nutrient and low oxygen levels and complex ecology. Several studies showed that E. faecalis was found more in cases of failed endodontic treatment than in cases with primary infections. Among all the reported cases with post-endodontic therapy pain and infection, it has been observed that E. faecalis is the most commonly found, with high prevalence values reaching up to 90%. Among all cases with primary endodontic infection, E. faecalis was more likely to be associated with asymptomatic cases than with symptomatic ones [3-5].

E. faecalis is an extensively evaluated biological indicator. Several laboratory studies tested the susceptibility of E. faecalis to endodontic treatment, which showed high resistance of E. faecalis to antimicrobial agents. Furthermore, E. faecalis can survive in very harsh environments, with poor nutrient supply and high alkaline pH reaching up to 11.5. The capacity of E. faecalis for growing as a biofilm on root canal walls and as a mono-infection in treated canals without synergistic support from other bacteria makes high resistance to antimicrobial agents a very resistance pathogen to root canal treatment [6-7].

Many studies that were found to discuss the association between E. faecalis with different forms of periradicular diseases. However, few studies have investigated the E. faecalis associated with endodontic treatment. Finally, the aim of this review was to review all recently published studies concerning Enterococcus faecalis as a dental root canal pathogen that causes endodontic failure.

## Review

Material and methods

This review has been performed meeting the guidelines of Preferred Reporting Items for Systematic Reviews (PRISMA).

The review question

The goal of this systematic review was to answer the following question:

“What is the effect of Enterococcus faecalis on endodontics treatment and what are the available treatment options to reduce the amount of E. faecalis during root canal treatment?”

Literature search

The literature search was made in two electronic databases (PubMed and Google Scholar) with MesH words (or/and) in different combinations as the following: ("Endodontic Infections" OR "Endodontic Pathogens," "Microbiology," and "Enterococcus Faecalis") taking into consideration the question of our review. The search was formulated in December 2019 and then updated in February 2020. Relevant articles had been identified and selected by scanning the titles and introduction of the included articles by the reviewers. For those articles that met the eligibility criteria, full-text articles were retrieved and downloaded in two ways. Free articles were downloaded directly from the database while restricted articles were downloaded by the institutional access of the library of King Abdulaziz University. Thereafter, study design and research were extracted from each retrieved article. For those articles that did not meet the main idea of our review, they were reviewed again and the final decision was made as to whether is was relevant or not.

Inclusion Criteria

1. Any research paper published in the English language

2. Articles published for a period of 10 years from 2009 - 2019

3. Studies conducted on human and animal subjects

Exclusion Criteria

1. Articles that illustrated the clinical relevance of Enterococcus faecalis in different dental specialties, excluding endodontics

2. Articles that described the different types of microorganisms in the root canal system excluding Enterococcus faecalis

Critical Appraisal

According to the eligibility criteria and PRISMA guidelines, included articles were analyzed independently by both reviewers. Any controversy between both reviewers was solved by a conversation between the two authors until the agreement was achieved.

Data Extraction and Presentation

**Figure 1:**
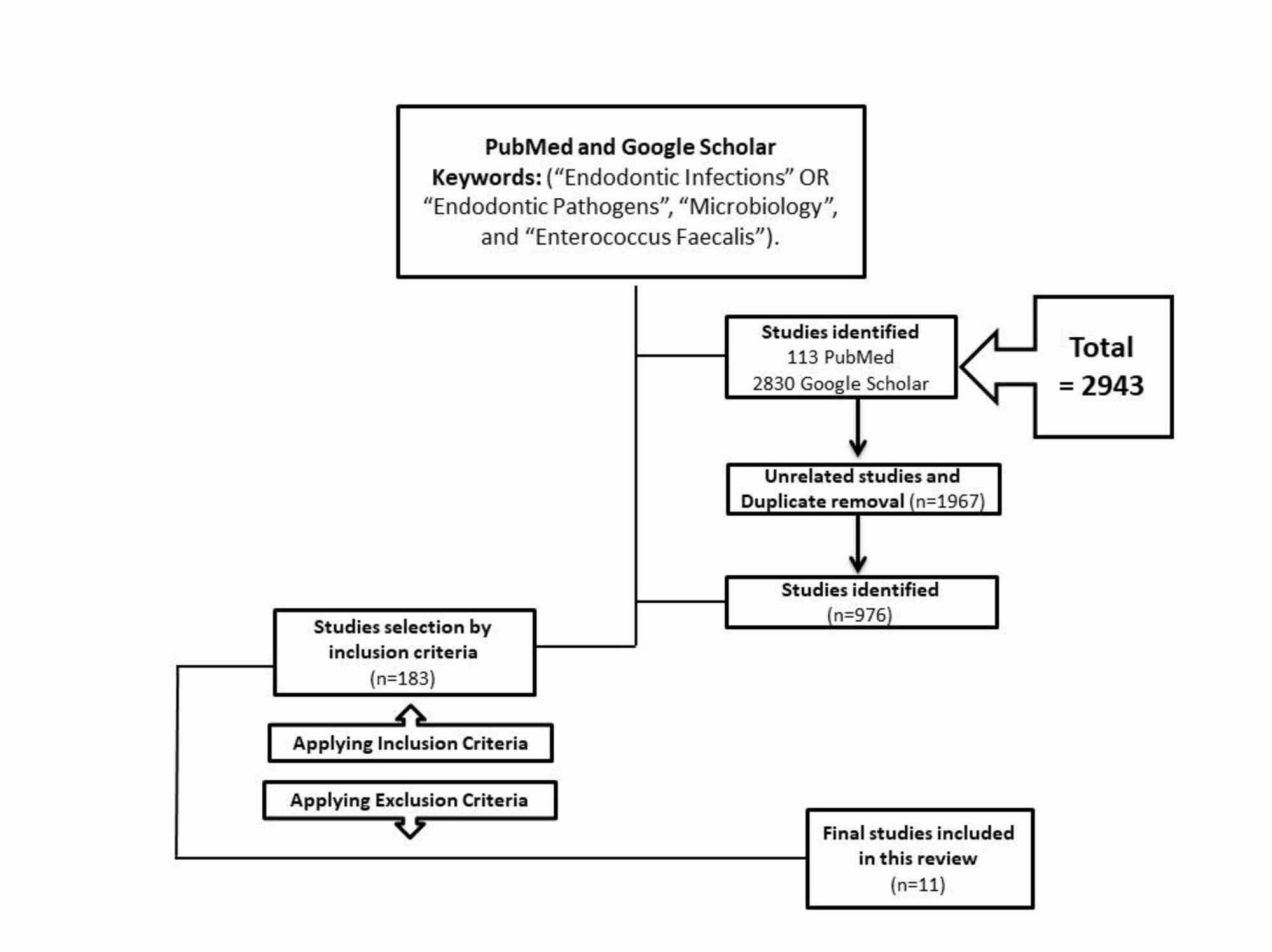
Flow chart showing the strategy used in this systematic review

Using the search keywords and MeSH words, two electronic databases (Google Scholar and PUBMED) were searched. The search strategy yielded a total of 2943 studies, in which 1967 articles were either irrelevant or duplicated. Among the relevant 183 studies, only 11 studies met the eligibility criteria and were included in this systematic review. The flow chart of the strategy search for this systematic review has been summarized and presented in Figure 1.

Results

The search was completed with only 11 studies that met the inclusion criteria. These 11 articles studied different aspects of Enterococcus faecalis, including its prevalence, characteristics, resistance mechanisms, express survival genes, and treatment. Most of the included studies conferred the high prevalence of E. faecalis within the root canal systems. Furthermore, it presented the ability of E. faecalis to affect the size of the periapical lesion and the microbial load within the root canal systems during the endodontic treatment. Additionally, it explained the ability of E. faecalis to survive by expressing some survival genes and using alternative metabolic pathways. All included studies were summarized in Table 1.

**Table 1:** Summary of all included studies in this systematic review

Authors/Study Design	Year	Main Conclusions
Endo M, et al. Brazil (In-vivo study)	2013	The great majority of taxa found in post-treatment samples were Gram-positive bacteria
Pereira RS, et al. Brazil (In-vivo study)	2017	Further studies are necessary to elucidate the role of these microorganisms in endodontic treatment failures.
Rôças IN, et al. Brazil (In-vivo study)	2012	The findings call into question the status of E. faecalis as the main pathogen and suggest that other species can be candidate pathogens associated with persistent/secondary endodontic infections.
Schirrmeister JF, et al. Germany (In-vivo study)	2009	In all teeth with Parvimonas micra and Dialister invisus, F. nucleatum and S. moorei were found. Moreover, members of additional different genera were detected delivering bacterial compositions that have been not described yet.
Jhajharia K, et al. Malaysia (Review)	2015	The most common endodontic infection is caused by the surface-associated growth of microorganisms.
Narayanan LL, Vaishnavi C. India (Review)	2010	The well-filled root canal offers the microbial flora a small, dry, nutritionally limited space. Thus, we should obtain a better understanding of the characteristics and properties of bacteria and their biofilms along with the environmental changes, to enhance success.
Del Fabbro M, et al. Italy (Review)	2014	The picture emerging from this review is that extraradicular infection is likely a multifactorial disease that requires further systematic investigation using standardized techniques.
Ran S, et al. China (In-vitro study)	2015	A number of the regulated genes may be useful candidates for the development of new therapeutic approaches for the treatment of E.faecalis infections.
OH Donmez, et al. Turkey (In-vitro study)	2019	The data of the induced and non-induced fabclavine promoter exchange mutants clearly show that fabclavine derivatives are bioactive compounds responsible for the bactericidal effect.
Lee D, et al. Korea (In-vitro study)	2019	These results suggest that phage HEf13 has the characteristics of a lytic phage and is a potential therapeutic agent for the treatment or prevention of E. faecalis-associated infectious diseases.
Ghorbanzadeh A, et al. Iran (Ex-vivo study)	2020	All three disinfection methods were effective for the partial elimination of E. faecalis biofilm. But conventional chemomechanical debridement + light-activated disinfection (CCMD + LAD) was significantly more efficacious in decreasing both mature and immature biofilms.

Discussion

This systematic review was compiled to assess the efficacy of E. faecalis when compared to a variety of other available microorganisms to assess the efficiency of the methods to remove these organisms from the root canal system as well as the periapical tissue. The 11 studies analyzed the E. faecalis cells or biofilms in different aspects and were conducted in the last 10 years.

Prevalence of E. faecalis

Various studies have analyzed the composition of the root canal system of previously treated teeth with a persistent apical lesion. Studies showed different results regarding the predominant species in failed endodontically treated cases. Pinheiro ET et al. emphasize that E. faecalis was the most frequently isolated bacteria from the root canal systems (45.8%) in previously treated cases [8]. Siqueira and Roças and Sedgley et al. reported similar results [9-10]. They observed the prevalence of E. faecalis was 77% and 79.5%, respectively, using the polymerase chain re­action (PCR). Furthermore, Sedgley et al. also detected that the prevalence of E. faecalis was significantly higher in retreatment cases (89.6%) than in primary infection (67.5%) [10]. However, four studies in this review discussed the presence of E. faecalis in the root canal system but in smaller percentages, as follows: 13.33%, 11.6%, and 12% [11-13]. In addition, the small percentages of E. faecalis detected confirmed that E. faecalis is not the main microorganism responsible for endodontic failure as compared to other studies. Even so, it is almost always present but in low per­centages [11-13]. Recent studies do not consider E. faeca­lis as the main microorganism associated with endodontic treatment failure. Therefore, in 2013, Endo et al. reported that Par­vimonas micra was the most prevalent microorganism in most of the cases (24%) [11]. Schirrmeister et al. also detected that Solobacterium moorei (33% of all teeth) and Fusobacterium nucleatum (33% of all teeth) were the most frequently found bacteria followed by Par­vimonas micra [14].

Resistance Mechanisms of E. faecalis

In our review, two studies discussed the resistance mechanisms of E. faecalis [15-16]. E. faecalis is an anaerobic, gram-positive, facultative coccus that causes opportunistic infections. It possesses many survival mechanisms to live in unfavorable conditions, such as to grow an environment with low oxygen, at high pH, at a wide range of temperatures between 10° and 60°, at high salinity or in a poorly nutrient environment [3-4,15-16].

Many studies have been conducted to find out the mechanisms that allow this pathogen to resist the disinfection measures during endodontic treatment. Narayanan LL and Vaishnavi C discussed the resistance mechanisms of E. faecalis to live in the presence of intracanal medication, such as calcium hypochlorite solutions and irrigants, and to make it able to escape those disinfection measures [16]. E. faecalis can use fluid in periodontal ligament as nourishment and form biofilms as protection against host resistance and disinfecting agents. Adding to those mechanisms, the ability of E. faecalis to develop antibiotic resistance, especially to erythromycin and azithromycin and to penetrate dentinal tubules and attached to collagen [8,16].

Characteristics of E. faecalis

In this review, only one study discussed some of the characteristics of this microorganism, which are summarized as follow: the capability of E. faecalis to survive in up to 6.5% concentrated sodium hypochlorite (NaOCl), sodium dodecy­lsulfate, hydrogen peroxide, heat, hyperosmolarity, ethanol, and both acidity and alkalinity [17]. Adding to these characteristics, E. faecalis can establish extra radicular infection by secreting toxins directly or through the induction of inflammation indirectly. Additionally, it can gain and transfer extrachromosomal elements and encoding virulence traits, which help colonize and compete with other bacteria, resist host defense mechanisms, and produce pathological changes. Furthermore, E. faecalis can make a well-organized biofilm that can resist the healing process. It can induce hydroxyapatite precipitation in a mature biofilm to form a calcified biofilm [17].

In a study done in 2006 by Stuart C et al., they added to the characteristics of E. faecalis the capacity of this microorganism to use serum from dentin and the periodontal ligament (PDL) as a source of nutrition [18]. As a result, this serum ensures the survival of E. faecalis and allows the bacteria to adhere to and invade the dentinal tubules [19]. Two authors discussed in a review study, the virulence factors of E. faecalis to establish endodontic infection and the induction of periradicular inflammatory response [20]. The most important virulence factors are aggregation substance, bacterial surface adhesins, sex pheromones, lipoteichoic acid, the production of extracellular superoxide, and the release of two important lytic enzymes: gelatinase and hyaluronidase. The aggregation sub­stances are plasmid-encoded adhesive substances, which support the exchange of the plasmid between the do­nor and recipient bacteria during the conjugation process between the bacteria. They also promote the binding between E. faecalis and various eukaryotic cells, enforce the adhesion of the bacteria to types I and IV collagens, and act as a protective factor against host neutrophils. Possessing these aggregation substances makes microorganisms such as E. faecalis qualified enough to stimulate the release of α tumor necrosis factors (TNF-α) by macrophages and the release of γ interferon (INF-γ) and β tumor necrosis factors (TNF-β) as a result of the proliferation of T cells induced by the bacteria [20]. As a consequence, the release of TNF will lead to bone resorption while the release of INF-γ will stimulate the secretion of more hydrogen peroxide and superoxide anions, resulting in damage to the cells and tissues. On the other hand, surface adhesins add to the virulence of E. faecalis by adhering the bacteria to many essential substances, such as abiotic surfaces (to form biofilm), other species of bacteria (for exchanging genes and nutrients), collagen fibers, human serum, and dentinal tissues [20].

The Express Survival Genes of E. faecalis

In this systematic review, only one study discussed how E. faecalis can survive in specific conditions [21]. A study by Ran S et al. explained the gene regulation responsible for the adaption process of these bacteria to alkaline stress conditions [21]. In pH 10, E. faecalis expresses 613 specific genes, out of which 211 genes were found to be up-regulated and 402 genes were down-regulated. The majority of over-regulated genes related mainly to genes encoding amino­acids and nucleotides transport and metabolism, which means that E. faecalis is capable of using some amino acids as carbon and energy sources, in high alkaline conditions, to encourage the biosynthesis of pyrimidine, resulting in an increase of bacterial viru­lence. On the other hand, the down-regulated genes are genes involved in the metabolism of carbohydrates and amino acids [21].

The glycolytic pathway of E. faecalis is affected during growth in alkaline conditions, leading bacterial cells to use alternative metabolic pathways to use less preferred energy sources such as fructose and mannose. Adding to the previous gene regulation mechanism, E. faecalis tend to synthesize stress-induced proteins in order to survive in some environmental stress. Although protein production can be blocked with chloramphenicol, this blockage had no effect on the survival of cells, which indicates that the production of stress-induced protein has a less important role for E. faecalis survival at a high pH [22]. The proton pump has an important role in regulating pH inside the bacterial cell to survive. When the pH be­came very alkaline due to Ca(OH)_2_ application, this will activate the proton pump and, as a consequence, the transport of protons into the cell to decrease the alkalinity of the cytoplasm. However, this pump will function well until it becomes saturated at a pH of 11.5. Then, the pump will stop working, resulting in cell death [22].

Treatment Options Against E. faecalis

In this systematic review, three studies discussed some treatment options to reduce or remove E. faecalis from the root canal system and the periradicular area [23-25]. Antibiotic medications were widely used for the treatment and prevention of E. faecalis caused by many pathogenic bacteria. In a study conducted by Donmez Ozkan H et al., in 2019, they found that fabclavine is an antimicrobial agent with a strong antibacterial effect against E. faecalis [23]. In this study, they found that fabclavine-rich supernatant was highly effective against broad strains of E. faecalis with multidrug resistance when used as an intracanal medicament. Other studies discussed novel treatment techniques using newly isolated phages for targeting E. faecalis strains from the oral cavity. Lee D et al. found that phage HEf13 has high lytic activity against human dentin, which suggests the effectiveness of using phage HEf13 as a dental therapeutic agent against E. faecalis-related apical periodontitis [24]. A recent study conducted by Ghorbanzadeh A et al. in 2020 found three effective disinfection methods for the partial elimination of E. faecalis biofilm [25].

Overall, this systematic review showed a strong trend toward supporting the role of E. faecalis in the failure of endodontic treatment. Furthermore, E. faecalis has specific characteristics that enable it to escape chemomechanical instrumentation during root endodontic treatment. These characteristics can be outlined as the following: ability to form biofilms and colonize in remote unreachable areas away from the main canals, such as accessory canals, apical deltas, and isthmuses, to be protected by residual tissue, dentinal tissues, human serum, and dead cells that reduce the effect of antimicrobial means. In addition, E. faecalis uses different mechanisms to survive in harsh environments. Those mechanisms include activating some survival genes, using alternative metabolic pathways, living in an area with high sources of a nutrient, and possessing bacterial synergism and aggregation capacity [26].

## Conclusions

In this systematic review, most studies give prominence to E. faecalis as the main pathogen responsible for the failure of endodontic treatment. E. faecalis have mechanisms to survive in different environments, for example, its ability to resist different measures of disinfection, to create a biofilm, to live in areas unreachable to the chemomechanical debridement of a root canal, and the synergistic reaction of different strains. Thus, clinical trials are required to provide valuable information about novel microbial detection methods to increase knowledge of the microbial species associated with endodontic infections and how to reduce those microorganisms in the root canal system.
